# Hardening of Cobalt Ferrite Nanoparticles by Local Crystal Strain Release: Implications for Rare Earth Free Magnets

**DOI:** 10.1021/acsanm.2c03161

**Published:** 2022-09-21

**Authors:** Beatrice Muzzi, Elisabetta Lottini, Nader Yaacoub, Davide Peddis, Giovanni Bertoni, César de Julián Fernández, Claudio Sangregorio, Alberto López-Ortega

**Affiliations:** † Department of Biotechnology, Chemistry and Pharmacy, University of Siena 1240, I-53100Siena, Italy; ‡ ICCOM−CNR, I-50019Sesto Fiorentino, Italy; § Department of Chemistry “U. Schiff”, University of Florence and INSTM, I-50019Sesto Fiorentino, Italy; ∥ IMMM, Université du Mans, CNRS UMR-6283, F-72085Le Mans, France; ⊥ Department of Chemistry and Industrial Chemistry, University of Genoa, I-16146Genova, Italy; # ISM−CNR, I-00015Monterotondo Scalo, Italy; ∇ CNR−Istituto Nanoscienze, I-41125Modena, Italy; ○ IMEM−CNR, I-43124Parma, Italy; ◆ Departamento de Ciencias, Universidad Pública de Navarra, E-31006Pamplona, Spain; ¶ Institute for Advanced Materials and Mathematics, Universidad Pública de Navarra, E-31006Pamplona, Spain

**Keywords:** cobalt ferrite, magnetic nanoparticles, microstrain, geometrical phase analysis, solvent-mediated annealing, coercivity

## Abstract

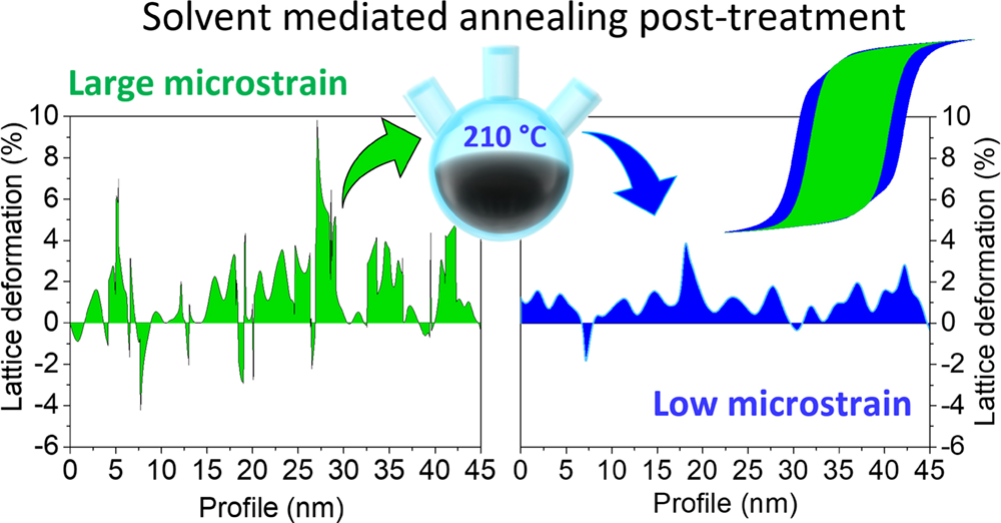

In this work, we demonstrate that the reduction of the local internal stress by a low-temperature solvent-mediated thermal treatment is an effective post-treatment tool for magnetic hardening of chemically synthesized nanoparticles. As a case study, we used nonstoichiometric cobalt ferrite particles of an average size of 32(8) nm synthesized by thermal decomposition, which were further subjected to solvent-mediated annealing at variable temperatures between 150 and 320 °C in an inert atmosphere. The postsynthesis treatment produces a 50% increase of the coercive field, without affecting neither the remanence ratio nor the spontaneous magnetization. As a consequence, the energy product and the magnetic energy storage capability, key features for applications as permanent magnets and magnetic hyperthermia, can be increased by ca. 70%. A deep structural, morphological, chemical, and magnetic characterization reveals that the mechanism governing the coercive field improvement is the reduction of the concomitant internal stresses induced by the low-temperature annealing postsynthesis treatment. Furthermore, we show that the medium where the mild annealing process occurs is essential to control the final properties of the nanoparticles because the classical annealing procedure (*T* > 350 °C) performed on a dried powder does not allow the release of the lattice stress, leading to the reduction of the initial coercive field. The strategy here proposed, therefore, constitutes a method to improve the magnetic properties of nanoparticles, which can be particularly appealing for those materials, as is the case of cobalt ferrite, currently investigated as building blocks for the development of rare-earth free permanent magnets.

## Introduction

Magnetic anisotropy and coercive force are key properties of magnetic materials, which, together with remanence and saturation magnetization, define their applicability in many technologies, such as magnetic hyperthermia, data storage, and permanent magnets.^1−3^ The reduction of the size down to the nanoscale is a well-established approach for the improvement of these properties in many traditional magnetic materials, such as spinel ferrites, hexagonal ferrites, or metal alloys.^4−6^ Matter at the nanoscale, indeed, exhibits exotic morphologies, structures, and properties which are intrinsically correlated with their metastable nature and arise from the different competitive and intertwined energy contributions coming from the dominant surface, size effects, the composition, and the chemical state.

Accordingly, the demanding requirements of technological applications have fueled the search for suitable strategies to improve the magnetic properties by fine controlling the morpho-structural features. In this framework, iron-based oxides, such as spinel ferrites or hexagonal ferrites, have demonstrated their high magnetic versatility through the control of their composition as well as of their shape and size.^7,8^ In addition, an extra degree of freedom is provided by the combination of diverse iron-based magnetic phases in exchange-coupled bi-magnetic nanosystems.^9,10^ Nevertheless, most of these strategies must cope with the intrinsic defectiveness which characterizes nanomaterials, arising from the constraints imposed during the synthesis to limit their growth to the nanoscale. Most of the studies reported so far focus on the modification of the surface of the nanoparticles (NPs) through reconstruction of the spin disordered shell,^11,12^ the increase of the surface anisotropy contribution,^13−15^ or by exchange-coupling.^16−18^ More recently, attention has focused also on the core spin structure.^19^ As an example, the synthesis of defect-engineered iron oxide NPs has been shown to be effective in boosting the hyperthermic efficiency of iron oxide NPs,^20^ as well as the permanent magnet properties in cobalt ferrite particles.^21^ Local distortions of the ligand field, dislocations, vacancies, antiphase boundaries, or the coordination distortions at the surface sites can indeed drastically affect the magnetic anisotropy of the final material.^21−23^

Thermal treatment is a conventional strategy to tailor the properties of the NPs, driving them to the minimum free energy configuration by extended changes of their morphological and chemical–physical properties through thermally driven atomic diffusions, crystal reconstruction, and shape modifications. However, the annealing can give rise to nucleation and growth of the particles, favoring the interparticle aggregation, reduction of the lattice defects, and charge-cation changes, mainly when air annealing is performed.^24^ Because all these effects are size-dependent and strongly intertwined, the tailoring of individual properties, such as the magnetic anisotropy, while keeping other structural and magnetic properties unaltered, is hard to be achieved.

Conversely, here, we demonstrate how choosing a mild thermal solvent mediated annealing, it is possible to release the internal stress without affecting the lattice defects, leading to an unprecedent final increase of the total magnetic anisotropy, without affecting any other structural or magnetic parameter (neither saturation, nor remanence magnetization). The validity of such an approach has been proved on cobalt ferrite (Co_*x*_Fe_3–*x*_O_4_) NPs of ∼30 nm prepared by thermal decomposition in benzyl ether. Cobalt ferrite is particularly suited to this aim because of its large cubic magneto-crystalline anisotropy, which is responsible for its high *H*_C_, the moderately high saturation magnetization, the large magnetostriction, high stability,^25,26^ and the easiness of preparation in the form of NPs with a controlled size and shape.^8^

The analysis of the effect of the solvent-mediated annealing at variable temperatures between 150 and 320 °C on the structural and magnetic properties clearly shows that, when low temperatures are considered (up to 210 °C) the thermal treatment causes the reduction of the nonuniform crystal lattice microstrain accumulated during the NP growth, increasing the *H*_C_, the energy product (*BH*)_max_, and the total magnetic energy of the nanomaterial. Moreover, we prove the crucial role of the solvent, compared to classical annealing procedures, to induce the effect.

## Results and Discussion

Octahedral ferrite NPs of composition Co_0.4_Fe_2.6_O_4_, as determined by energy-dispersive X-ray fluorescence (EDXRF) analysis (Supporting Information, Table S1), with average edge size, *l*, of 32(8) nm and narrow particle size distribution (σ < 20%), were synthesized by thermal decomposition of metal–organic precursors in the high-boiling point solvent benzyl ether, as described in ref (8) (sample **CFO**). Afterward, the as-prepared NPs were dispersed in high boiling solvents (benzyl ether or octadecene) with the help of oleylamine (OAm) and oleic acid (OA) as surfactants and annealed under an inert atmosphere at different temperatures, 150, 210, 270, and 320 °C. In the following, the samples will be denoted as **CFO#** where # corresponds to the annealing temperature. In addition, a fraction of the as-prepared sample was annealed in an oven at 210 °C under an inert atmosphere, to compare the effect of the heating media in the thermal treatment (sample **CFO210-oven**).

**Figure 1:**
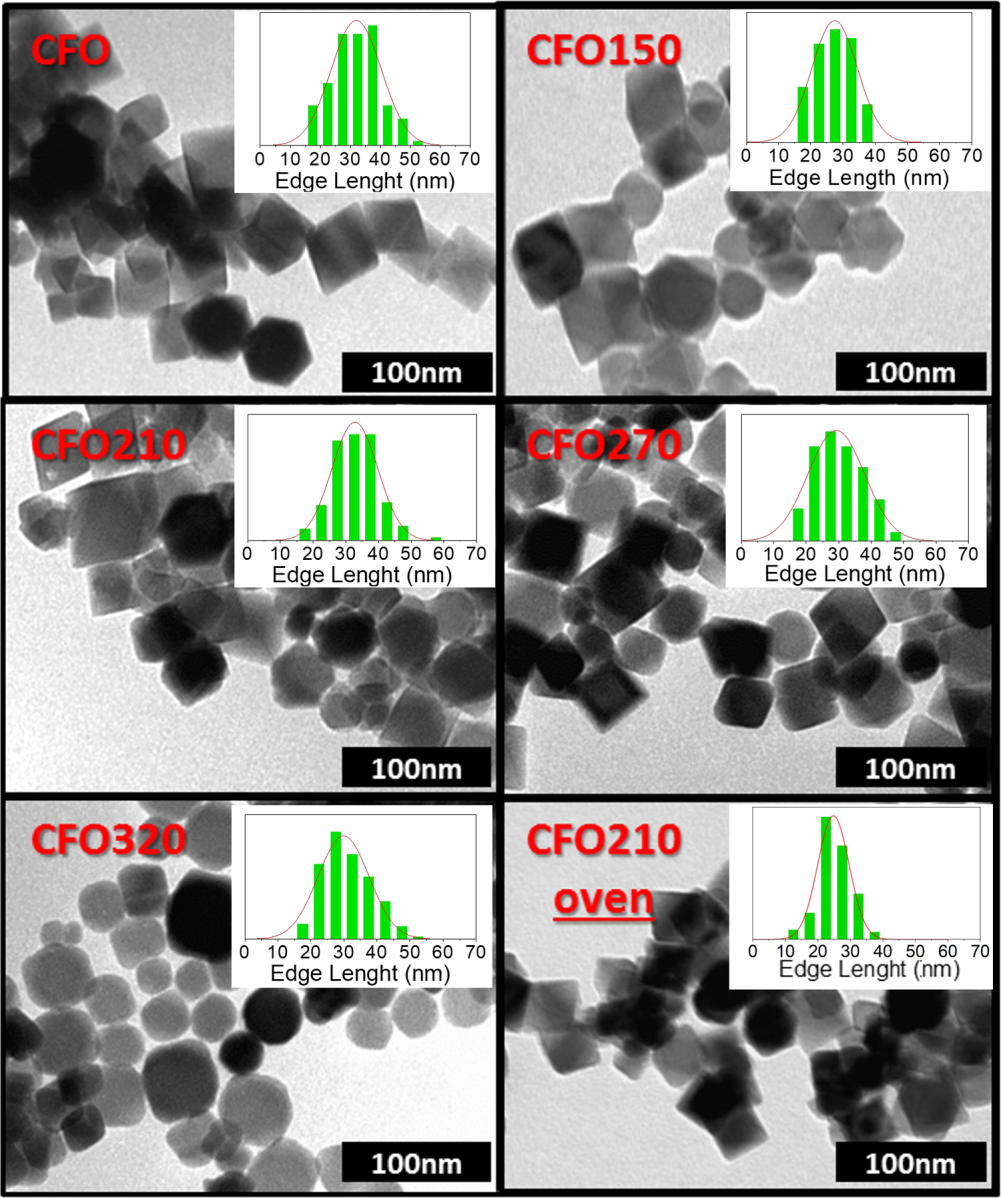
Selected TEM images and corresponding particle edge length histograms for as-prepared and annealed nanoparticles.

Figure 1 depicts representative bright-field, low-magnification, transmission electron microscopy (TEM) images of the as-prepared and annealed NPs, with the corresponding particle size histograms. **CFO** comprises octahedral shaped particles, as expected for cobalt ferrite grains larger than 20 nm.^27^ The annealing process does not modify the particle size nor the size distribution of the system, which maintains a narrow deviation, lower than 20% (see Table 1). Moreover, the thermal treatment does not have any effect on the shape of the NPs when the heating temperature is increased up to 270 °C, independently of the heating process used (oven or solvent mediated annealing). Conversely, at higher temperatures (320 °C) the thermal process has a smoothing effect on particle edges changing the NP shape in truncated octahedrons or cubes. Such modification can be easily explained by the higher reactivity of the NP corners, which are the first part of the crystal affected by Ostwald ripening, induced by the relatively high temperatures used in the annealing process.^28^

**Table 1:** Summary of the Annealing Temperature and Structural Data Obtained from TEM and XRD Analyses^a^

samples	annealing temperature (°C)	TEM	XRD		
		*l* (nm)	*a* (nm)	microstrain	crystal size (nm)
**CFO**		33(2)	0.8398(1)	7.0 × 10^–4^	45(2)
**CFO150**	150^*(sol.)*^	29(2)	0.8404(1)	1.3 × 10^–5^	41(2)
**CFO210**	210^*(sol.)*^	33(2)	0.8406(1)	4.9 × 10^–7^	41(2)
**CFO270**	270^*(sol.)*^	29(6)	0.8404(1)	4.7 × 10^–6^	40(2)
**CFO320**	320^*(sol.)*^	30(2)	0.8401(1)	2.3 × 10^–4^	40(2)
**CFO210-oven**	210^*(pow.)*^	27(2)	0.8403(1)	1.0 × 10^–3^	40(2)

a TEM particle size was assessed by considering the edge length of cubes or octahedrons (*l*) and fitting the distribution to a lognormal function. *a* refers to the cell parameter for the cubic spinel structure. Lattice parameter, microstrain, and crystal size are obtained by Rietveld refinement of the experimental patterns. Uncertainties on the last digit are given in parentheses. Note that the error for microstrain has been assessed to the 10% of the calculated value.

High-resolution TEM (HRTEM) investigations performed on **CFO** and **CFO210** (see Supporting Information, Figure S1) confirmed that the two samples share the same morphology (octahedral shape) and have a highly ordered crystal structure along the entire NP. The detailed analysis of the fast Fourier transform (FFT) images revealed that the characteristic spots for the cobalt ferrite structure, observed in the as-prepared sample, **CFO**, are preserved after the annealing process (see Supporting Information, Figure S1). Finally, EDXRF measurements proved that the stoichiometry of the pristine sample (Co_0.4_Fe_2.6_O_4_) is maintained for all the samples, and thus no metal ion leaking occurs during the heating (Supporting Information, Table S1).

**Figure 2:**
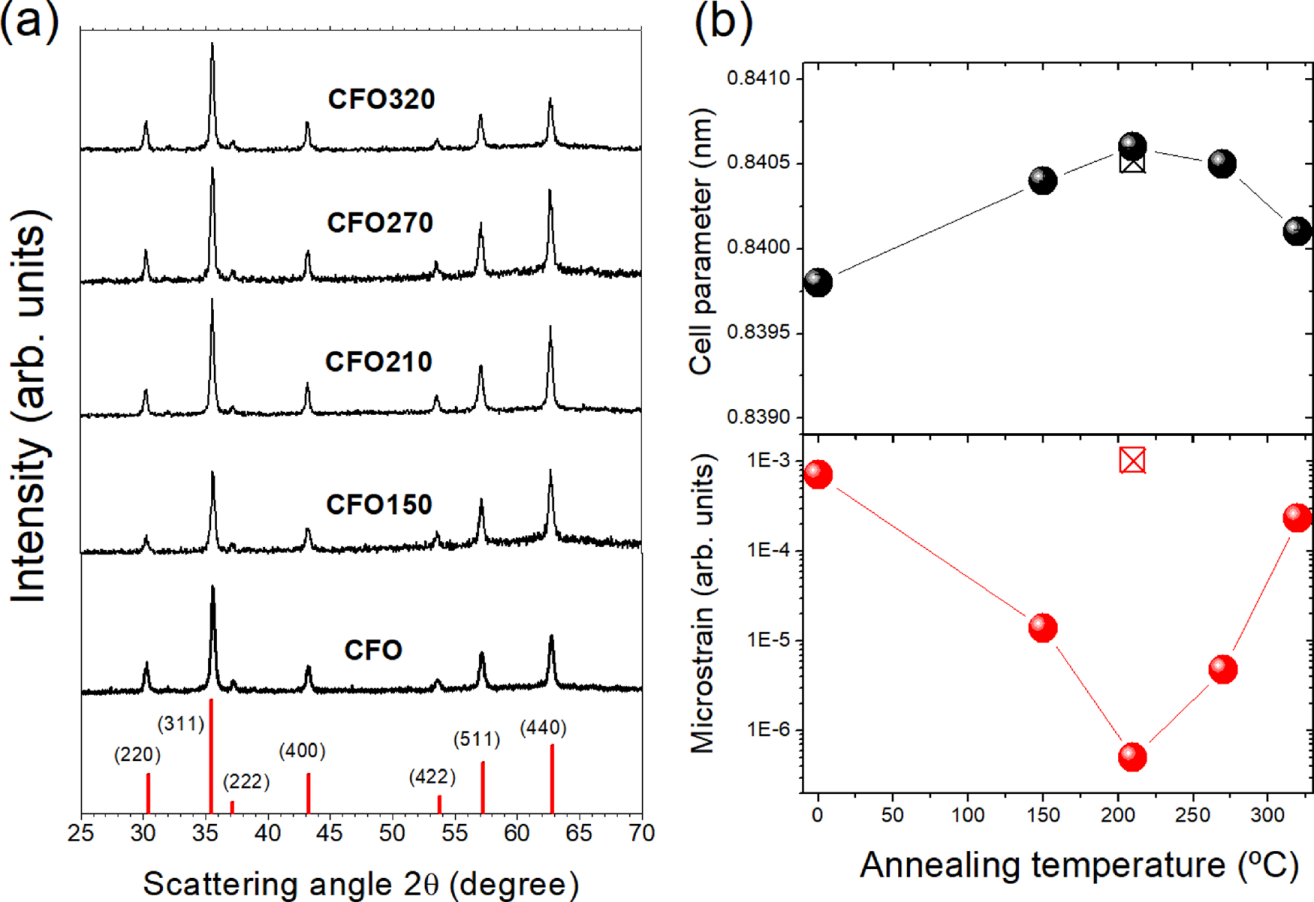
(a) XRD patterns for the as-prepared and annealed cobalt ferrite NPs (red bars refer to the position of the diffraction peaks for the cobalt ferrite crystal structure). (b) Cell parameter (top) and microstrain (bottom) dependence on the annealing temperature (black ballot box with X and red ballot box with X refer to the **CFO210-oven** sample). Error bars are not visible because they are smaller than symbols.

The X-ray diffraction (XRD) patterns indicate the presence of a single crystallographic phase (Figures 2a and S2), indexed to a cubic spinel structure (space group *Fd*3̅*m*, JCPDS PDF #221086). The calculated crystal size is consistent with that obtained from TEM images, indicating the growth of highly ordered single-crystal structures (Table 1). The cell parameter, *a*, for all the series of samples is in the range expected for nonstoichiometric cobalt ferrite, that is, Co_0.4_Fe_2.6_O_4_;^27,29^ however, as the annealing temperature is increased a nonmonotonic dependence is observed with a maximum for the sample annealed at 210 °C (**CFO210**). An opposite trend is displayed by the microstrain, estimated from the Rietveld analysis of the experimental pattern, although in this case the percentage change is much more pronounced (Figure 2b and Table 1) spanning three orders of magnitude between **CFO,** 7.0 × 10^–4^, and **CFO210**, 4.9 × 10^–7^. Both trends can be explained by the reduction of internal stresses created during the growth of the NPs, considering that a microstrain value below 1 × 10^–5^ indicates its quasi-complete disappearance.

The annealing treatment, indeed, permits the reduction of the intrinsic plastic deformations created during the growth of the nanocrystallites. These deformations are responsible for local distortion of lattice planes that gives rise to a nonuniform variation in the interplanar distances (i.e., microstrain).^30^ As the annealing temperature is increased, a progressively larger partial release of internal stress occurs, reducing the associated strain. However, when the temperature reaches 270 °C, the increased reactivity of the system in the annealing medium can slightly affect the cationic homogeneity along the NPs, modifying the cell parameter, with a consequent increase of the strain in the nanostructure.^31,32^ On the other hand, the sample annealed at 210 °C in the oven (**CFO210-oven**) has cell parameter and crystal size values consistent with those obtained for the solvent-mediated annealed samples, but the calculated microstrain is much larger. The different heating treatment environment has thus a different effect on the capability of the NPs of releasing the stress.

**Figure 3:**
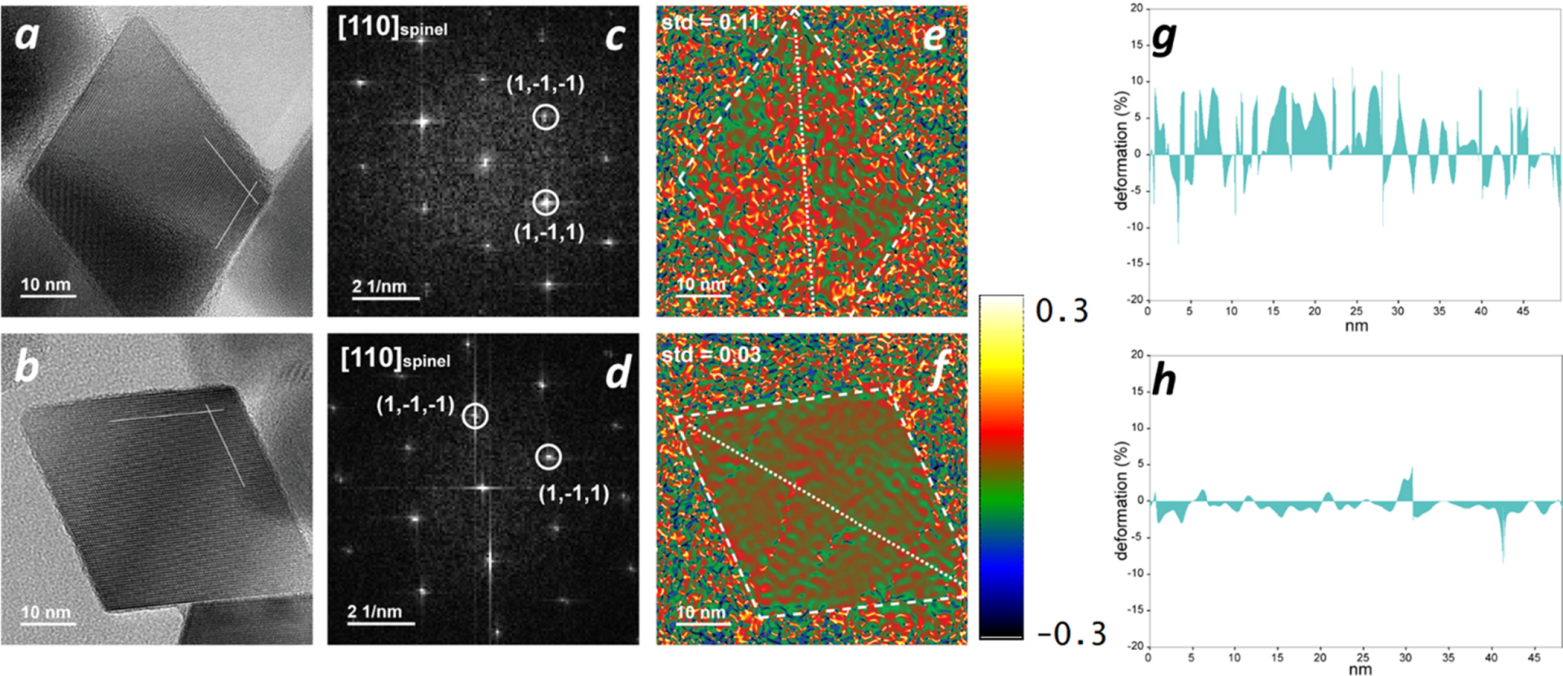
HRTEM images of a (a) **CFO** and a (b) **CFO210** NPs with FFT analysis (c,d) obtained from the NP in (a) and (b), respectively. The labeled spots are related to crystallographic planes that can be indexed as cubic spinel structure (*Fd*3̅*m*), in zone axis [110]. Strain maps ε_*xy*_ (symmetric shear) obtained by GPA analysis of the NPs (e) before and (f) after thermal annealing. The maps are calculated from (e,f) *g*_1_ = (1-1-1) and *g*_2_ = (1-11) reflections respectively, and plotted in the relative interval (−0.3, +0.3). Lattice deformation percentage profiles of (g) **CFO** and (h) **CFO210** measured along the diagonal of the NP (dotted white line), which allowed to estimate the relative displacement of the (111) planes with respect to the reference region (see the SI for further details).

These results are nicely supported by local analysis performed on a single NP (Figure 3) by geometrical phase analysis (GPA).^33^ The comparison between **CFO** and **CFO210** highlights that, after the thermal treatment, the displacement of the crystallographic planes in the 3D lattice is reduced. The strain maps were calculated for the (002) and (111) reflections (Figure S1) to estimate the displacement of these planes from their reference (i.e., average) positions in the spinel structure. The strain mapping clearly shows that after thermal annealing the lattice strain significantly decreases in comparison with the as-prepared samples, the lattice deformation being less than ca. 2% for sample **CFO210** (Figure 3f). Conversely, no significant changes were distinguished for the (002) crystallographic plane before and after thermal treatment. Thus, the disorder in the as-prepared MNPs originates mainly from (111) planes in **CFO**, and then it is mitigated by the thermal treatment in **CFO210** (see the Supporting Information for more details).

**Figure 4:**
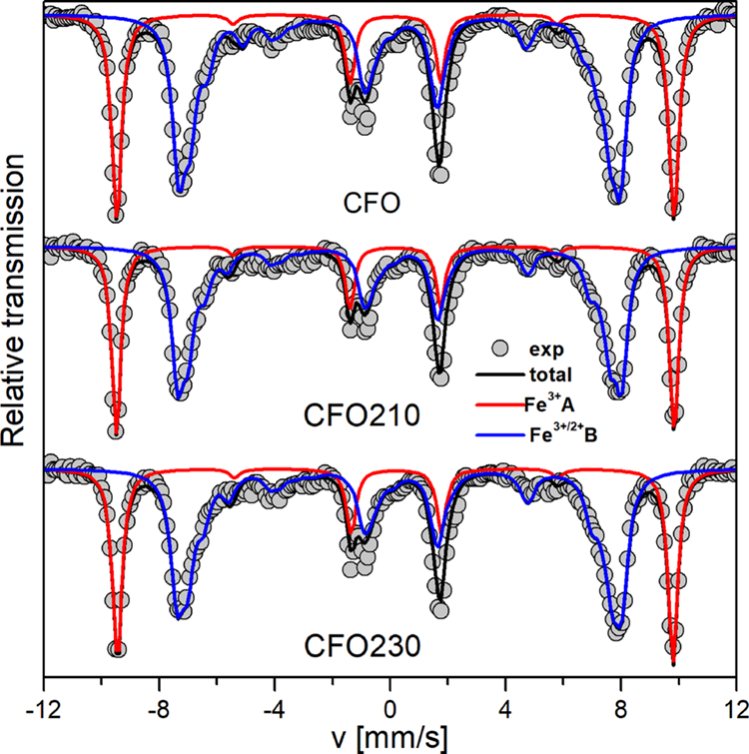
Mössbauer spectra measured at 12 K under 8 T magnetic field for **CFO**, **CFO210**, and **CFO320**. Gray dots and black solid lines are experimental and simulated total spectrum. Red and blue lines are the simulated sub-spectrum corresponding to A and B sites, respectively. Detailed fit of A and B sites is reported in the Supporting Information (Figure S3).

To investigate the evolution of the magnetic structure with the annealing temperature, ^57^Fe Mössbauer spectra under intense magnetic field (8 T) were recorded at 12 K on selected samples **CFO**, **CFO210**, and **CFO320** (Figure 4). For some details on the technique and on the fitting of spectra see the Supporting Information. The in-field spectra of the different samples are split into two main sub-spectra corresponding to the iron ions located at tetrahedral (A) and octahedral (B) sites, respectively.

It should be noted that while the refined values of the mean isomer shift, δ, (Table 2) correspond to those expected for Fe^3+^ in the A-site for the cobalt ferrite structure, slightly higher values are observed in B-sites, indicating the presence of some Fe^2+^. The presence of divalent iron cations has been also confirmed by the mean refined value of the hyperfine field, *B*_hyf_, which are lower than those of stoichiometric cobalt ferrite.^34−36^ The relative population of Fe in A and B sites is equal within the experimental error for all the samples, indicating that thermal treatment does not affect cation distribution.^34,37,38^ Moreover, the nonzero intensity contribution clearly indicates the presence of a noncollinear spin structure, showing a mean canting angle that, within the experimental error, is equal for all the samples.^38−40^

**Table 2:** Mean Isomer Shift (δ), Mean Quadrupole Shift (2ε), Mean Hyperfine Field (*B*_hyf_), Mean Canting Angle (θ), and Relative Amount of Fe in A and B Cavities Evaluated from Fitting In-Field Mössbauer Spectra Are Reported for Samples **CFO**, **CFO210**, and **CFO320**^a^

sample	site	δ (mm s^–1^)	2ε (mm s^–1^)	*B*_hyf_ (T)	θ (°)	Fe_A,B_/Fe_total_
**CFO**	Fe^3+^_A_	0.36(1)	–0.00(1)	52.0(2)	15	0.34(1)
	Fe^3+/2+^_B_	0.60(1)	–0.11(1)	51.2(2)	24	0.66(1)
**CFO210**	Fe^3+^_A_	0.35(1)	–0.00(1)	52.0(2)	14	0.34(1)
	Fe^3+/2+^_B_	0.60(1)	–0.15(1)	51.6(2)	23	0.66(1)
**CFO320**	Fe^3+^_A_	0.35(1)	–0.00(1)	51.8(2)	15	0.34(1)
	Fe^3+/2+^_B_	0.60(1)	–0.12(1)	51.2(2)	25	0.66(1)

a Uncertainties on the last digit are given in parentheses and the error for canting angle has been assessed to be 10°.

**Figure 5:**
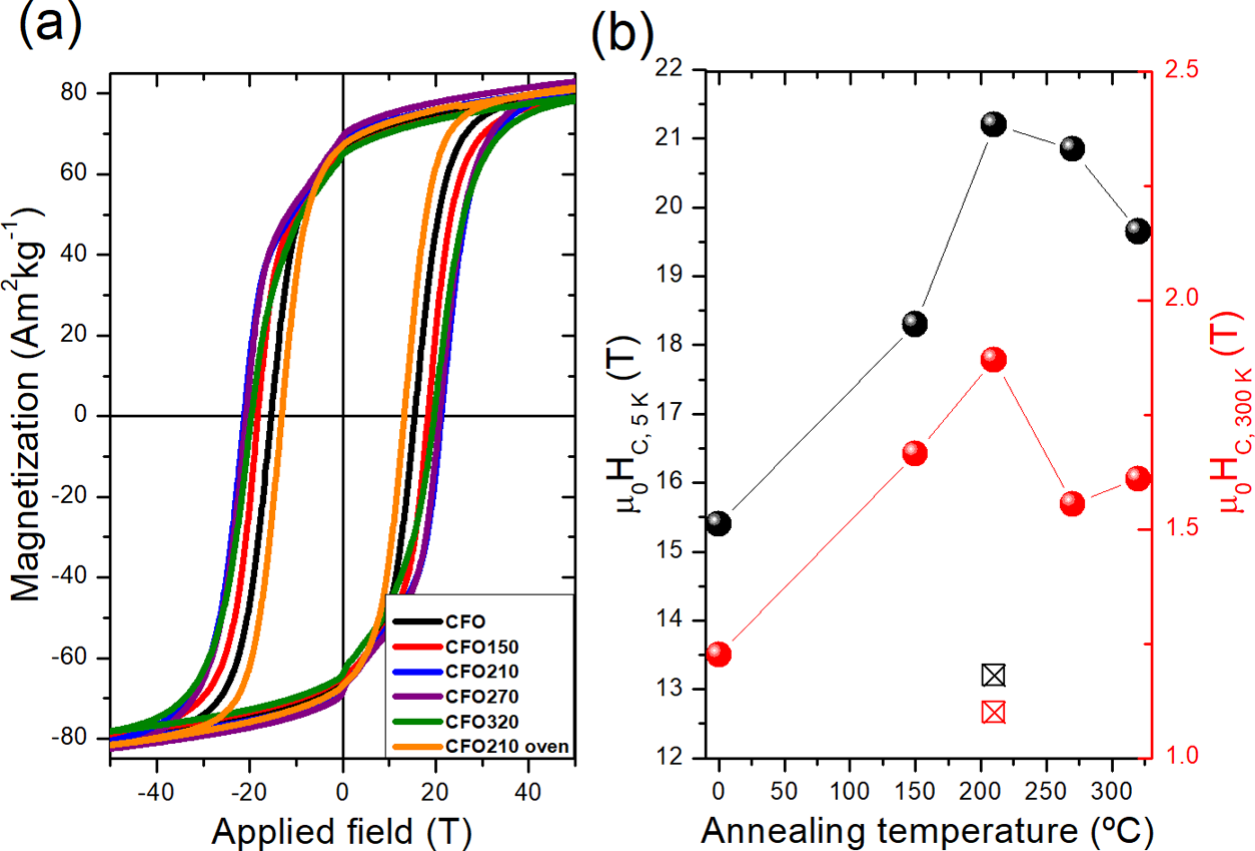
(a) Hysteresis loops for the as-prepared and annealed cobalt ferrite NPs measured at 5 K; (b) coercive field (μ_0_*H*_C_) dependence measured at 5 K (black) and 300 K (red) as a function of the annealing temperature (black ballot box with X and red ballot box with X refer to **CFO210-oven**).

The magnetic properties of the samples were investigated by measuring the hysteresis loop at 5 and 300 K. Low-temperature measurements (Figures 5 and S4) performed after a field cooling procedure show that the pristine material has μ_0_*H*_C_ of ∼1.5 T, a specific saturation magnetization value at 5 T (*M*_5T_) of ∼80 Am^2^ kg^–1^, and a reduced remanence value (*R* = *M*_R_/*M*_5T_) of 0.83 (Table 3). These values are in agreement with those expected for an assembly of randomly oriented, nonstoichiometric cobalt ferrite NPs of roughly 30 nm.^8,26,29^ Moreover, the comparison of the loops of the various samples demonstrates that the annealing procedure does not affect neither the saturation magnetization of the system nor the reduced remanence. This result confirms that thermal treatment does not force internal chemical reactions or large modifications of the crystal and spin structures of the system, as indeed already indicated by XRD and Mössbauer experiments. Conversely, the coercive field is largely modified by the annealing procedure and exhibits a nonmonotonic behavior with a maximum for **CFO210** corresponding to 2.12 and 0.19 T for 5 and 300 K, respectively, (Figure 5b), which is ca. 60% larger than **CFO**.

**Table 3:** Magnetic Properties of Cobalt Ferrite NPs Measured at 5 and 300 K: μ_0_*H*_C_ Coercive Field, *R* Reduced Remanence, *M*_5T_ Specific Saturation Magnetization at 5 T, *M*_S_ Extrapolated Saturation Magnetization, *K*_eff_ Effective Magnetic Anisotropy, and (*BH*)_max_ Room-Temperature Energy Product^a^

samples	5 K					300 K			
	μ_0_*H*_C_ (T)	*R*	*M*_5T_ (Am^2^ kg^–1^)	*M*_S_ (Am^2^ kg^–1^)	*K*_eff_ (MJ m^–3^)	μ_0_*H*_C_ (T)	*R*	*M*_5T_ (Am^2^ kg^–1^)	(*BH*)_max, 300 K_ (kJ m^–3^)
**CFO**	1.54	0.83	80	84	1.01	0.12	0.34	78	7.2
**CFO150**	1.83	0.82	79	83	1.23	0.17	0.48	78	8.8
**CFO210**	2.12	0.84	81	84	1.39	0.19	0.49	79	12.7
**CFO270**	2.09	0.84	83	86	1.35	0.16	0.48	80	12.0
**CFO320**	1.96	0.82	79	82	1.28	0.16	0.46	77	10.4
**CFO210-oven**	1.32	0.83	82	85	0.83	0.11	0.34	79	5.6

a Uncertainties on the last digit are given in parentheses. The errors for *M*_S_, *M*_5T_, and μ_0_*H*_C_ have been assessed to be the 5 and 2%, respectively, of the experimental values.

Interestingly, the μ_0_*H*_C_ trend well reproduces that observed for the cell parameter and microstrain. Therefore, by considering that neither chemical nor structural modifications occurred during the annealing, it can be argued that it is the reduction of the internal strain in the NPs that leads to a net increase of the coercivity of the sample. This behavior is contrary to that previously reported for submicron cobalt ferrite particles prepared by a ball-milling process,^41−44^ where the strong energy generated during the milling causes the proliferation of structural defects generating stress-induced anisotropy and pinning centers for domain wall motion, which increase *H*_C_.^44^ However, in the present case, thanks to the high crystallinity of the NPs the presence of this kind of stress anisotropy can be neglected. Moreover, the nanometric dimension of the NPs allows for excluding the presence of domain-wall driven switching, so that pinning centers cannot determine the magnetization dynamics of the system.^8,45^

Any attempt to rationalize this unexpected behavior requires taking into account the magneto-elastic energy, which adds a contribution to the total magnetic energy that, in a single domain particle, determines the reversal dynamics and the coercive field (inverse magnetostrictive effect).^46−48^ In the framework of the Stoner–Wohlfarth model, a strain, ε, induces, in random oriented particles, an additional uniaxial magnetic anisotropy, characterized by the magneto-elastic anisotropy constant, *K*_elas_ = 3/2λ_s_ε, where λ_s_ is the magneto-elastic constant. Because bulk cobalt ferrite has a cubic anisotropy and it is an anisotropic magneto-elastic material, λ_s_ should be written as λ_s_ = 1/5 (2λ_100_ + 3λ_111_), where λ_100_ and λ_111_ are the magnetoelastic constant along the <100> and <111> directions. We can first consider the effect of the stress release associated with the variation of the lattice parameter, σ = (*a*_CFO210_ – *a*_CFO_)/*a*_CFO_ = +9 × 10^–4^, where *a*_CFO_ and *a*_CFO210_ denote the cubic edge of the unit cell of **CFO** and **CFO210**, respectively, and the latter sample is assumed to have no residual strain (ε = 0). The equivalent strain ε is given by ε = *E*σ where *E* is Young’s modulus. Considering the values for *E* and λ_s_ reported in the literature for cobalt ferrite (*E* = 1.4 × 10^11^ N m^–2^, λ_100_ = −425 × 10^–6^, and λ_111_ = +163.7 × 10^–6^)^49,50^ and the experimental lattice parameter variation, we obtain *K*_elas_ = −1.4 × 10^4^ J m^–3^. This value is largely overestimated because it refers to a unidirectional strain, while the lattice variation is isotropic. Nevertheless, independent of its magnitude, *K*_elas_ provides a negative contribution along the same directions of the magneto-crystalline anisotropy (we remind that cobalt ferrite has positive cubic magneto-crystalline anisotropy, which makes the easy axes to lie along the <100> directions). Therefore, if this were the dominant term, the coercivity of **CFO** would be larger than that of **CFO210**, which is not the case.

On the other hand, the structural characterization evidenced that the release of microstrain occurs preferentially along the {111} planes. This microstrain is responsible of compressions and expansions of the interplanar distance with respect to the average value, which is also confirmed by the GPA maps and lattice deformation profiles. This positive and negative local strain will differently affect the energy profile for the reversal of the magnetization, creating exchange coupled regions where the local magnetic anisotropy is higher and others where it is lower than the unstrained sample. As an example, if fluctuations ε = ±7 × 10^–4^ along the <111> directions are considered, an additional contribution to the local magnetic anisotropy energy of +®2.2 × 10^3^ J m^–3^ is obtained, which is large enough to modify the reversal process of the magnetization (the bulk magneto-crystalline anisotropy is in fact 5 × 10^4^ J m^–3^). Moreover, lattice deformation profiles suggest that local lattice parameter fluctuations in the {111} planes are much higher than the average value obtained by XRD analysis used for this rough estimation (even larger than *K*_elas_ value previously evaluated). Therefore, strong magnetic anisotropy local fluctuations will be likely to occur in **CFO**, which can favor incoherent magnetization reversal mechanisms into the particles. These latter are characterized by a lower energy barrier than the pure coherent rotation process, which is presumably operative in **CFO210**, reducing the coercivity of the material. We can thus conclude that in the case of our single-domain cobalt ferrite NPs the improvement of the magnetic properties is driven by the reduction of the internal strains and of the nonuniform variations in the interplanar distances inside the NPs.

**Figure 6:**
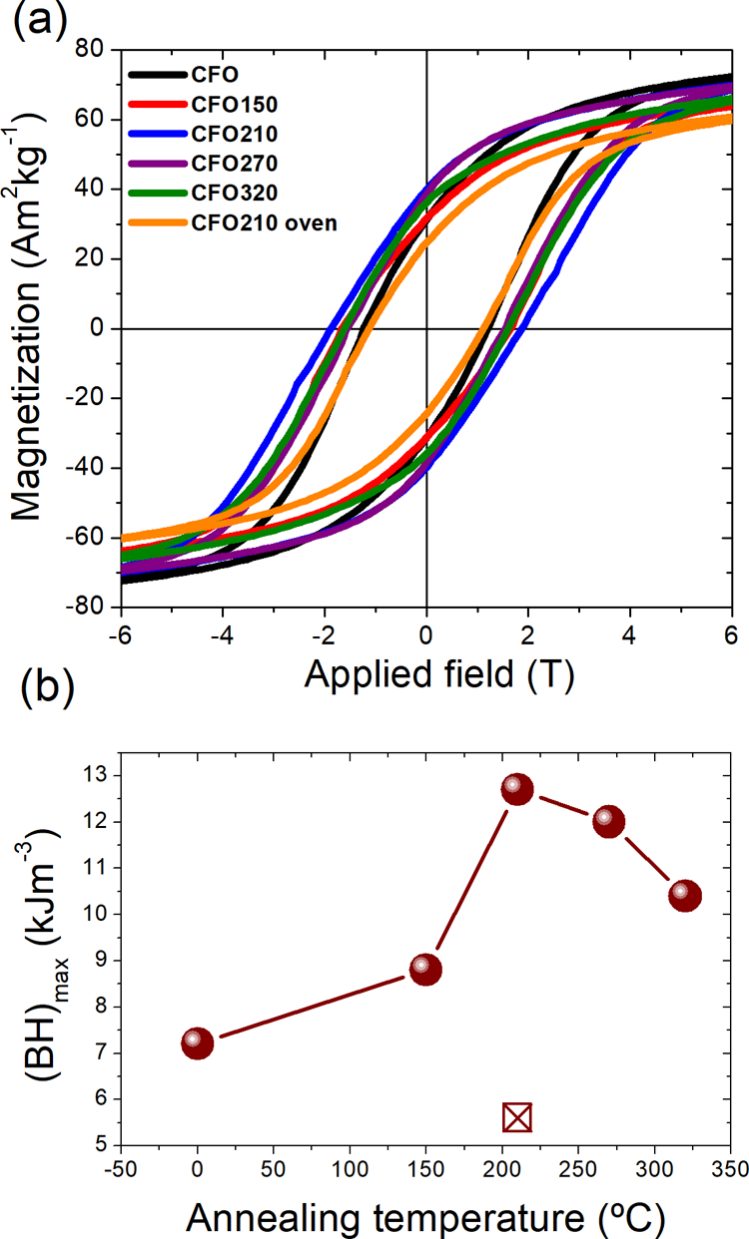
(a) Hysteresis loops for the as-prepared and annealed cobalt ferrite NPs measured at 300 K (enlargement of the low field region). (b) (*BH*)_max_ product as a function of the annealing temperature (black ballot box with X refers to the **CFO210-oven** sample).

To further evidence the impact of the thermal treatment on the magnetic properties, the maximum energy product (*BH*_max_) at RT, which is the figure of merit of a permanent magnet, is calculated as a function of the annealing temperature (see Figure 6).^51^*BH*_max_ is 7.2 kJ m^–3^ for **CFO** and then it varies with the thermal treatment temperature following the same nonmonotonic trend observed for the coercive field, with a maximum increase of more than 70% for **CFO210** (12.7 kJ m^–3^). Similarly, magnetic losses, which also depend on the area of the BH hysteresis loop, increase by the same percentage, from 131 kJ m^–3^ for **CFO** to 226 kJ m^–3^ for **CFO210,** respectively. This energy can be released as thermal energy after a complete hysteresis loop is completed, and thus, it defines the performance of the material as heat mediators in key technologies as tumor therapy by magnetic fluid hyperthermia^52−55^ and magnetic induction catalysis.^56−59^ These results further confirm that a controlled annealing procedure in a solvent-mediated medium allows for improving the capability of this material of storing energy.

It should be stressed that the described modification of NPs’ magnetic anisotropy is not simply related to a temperature effect, but also depends on the NP environment state (solid or liquid) where the heating process is carried out. As reported in Table 3 and Figure 5, indeed, when dried **CFO** cobalt ferrite NPs are annealed in an oven at 210 °C (**CFO210-oven**), they exhibit a reduced coercivity, not only with respect to NPs annealed at the same temperature in solvent (**CFO210**) but also to the starting sample. This effect can be attributed to the different NPs’ surface–surfactant interaction, which characterizes the two processes. Indeed, in a solid–liquid system, such as that of the solvent-mediated annealing, the surfactant adsorption is a dynamic equilibrium where the molecules are perpetually arriving at and leaving the surface.^60^ Thus, during the solvent-mediated annealing, the NPs’ surface is partially free to reduce the lattice strain with a decrease of the planes’ deformation percentage (Figures 4 and S1). Conversely, in the case of the oven-annealed powder, the organic capping layer forms a very stable thin layer around the NPs, which can hamper the complete release of the accumulated stress.^30^ On the other hand, the presence of a thin capping layer can also modify the NPs’ interactions. Indeed, Δ*M*_plots_ (Figure S5 in the SI) performed on **CFO**, **CFO210**, **CFO320**, and **CFO210-oven** have shown for all the samples a prevalence of demagnetizing (i.e., dipolar) interactions. **CFO**, **CFO210**, and **CF320** show comparable interactions strength, with an increase of Δ*M* field corresponding to the increase of MNPs’ magnetic anisotropy. Conversely, an evident increase of interparticle interaction strength had been observed in **CFO210-oven**.

Finally, it is worth stressing that the observed behavior is not occasional. To confirm the reproducibility of the methodology herein presented, another set of cobalt ferrite NPs (**CFO_2_#**), with average edge size, *l*, of 43(5) nm, was subjected to controlled solvent annealing treatment at variable temperature (Supporting Information, Figure S6). Even for **CFO_2_#** a net increase of the coercive field was observed for the NPs annealed in solution with a maximum at 210 °C. This result confirms that the solvent-mediated annealing is an effective and reliable pathway to control the magnetic properties of NPs, through the release of internal structural stress of the particles.

## Conclusions

The thermal decomposition approach has been proposed in the literature as an efficient route to prepare cobalt ferrite NPs with high-energy products. Nevertheless, although at a first sight this technique provides magnetic nanostructures exhibiting high structural and spin arrangement orders, we here demonstrated that postsynthesis mild treatment at controlled low temperature can be effective to significantly improve the energy product of the material. We indeed found that a solvent-mediated annealing treatment in mild conditions (ca. 200–300 °C) permits the reduction of the internal stresses generated during the nanocrystal growth, without affecting neither the morphology, nor the chemical composition, or the structure of the NPs.

A deep structural investigation pointed out that the as-prepared cobalt ferrite NPs display anisotropic lattice strain, the family planes passing through the tetrahedral sites, that is, {111}, being characterized by the highest lattice deformations. These local deformations produce fluctuations of the energy barrier for the magnetization reversal, favoring the onset of incoherent reversal processes. The solvent-mediated annealing treatment allows the release of the local internal strain and fosters the restoring of a pure coherent reversal process. Because the latter is associated with a higher energy barrier, the coercive field increases, being up to 50% higher than in the pristine sample. The effect was found to be maximum at 210 °C. The treatment at higher temperature (*T* > 300 °C), in fact, modifies the cationic homogeneity in the NPs, decreasing the cell parameter, with a consequent enhancement of the strain.

Furthermore, it has been shown that the medium where the annealing process occurs is essential to control the final properties of the NPs: in the classical annealing procedure performed on a dried powder, the stability of the organic layer surrounding the NPs does not allow the release of the lattice stress accumulated in the NPs, leading to the reduction of the initial coercivity; on the contrary, the solvent mediated treatment resulted in a long-range ordered crystal structure. It should be emphasized that the proposed post-treatment procedure can be envisaged to improve the magnetic properties of a broad range of nanomaterials, as, for example, soft ferrites, whose magnetic properties are critically strain-dependent.

On the other hand, this work reminds us that magnetic NPs should be always considered as metastable structures, which, being thermodynamically less favored than the corresponding micro and macrosized materials, naturally evolve during each postsynthesis treatment. Therefore, care must be taken whenever the magnetic behavior of complex multicomponent nanoheterostructures, obtained by multistep procedures, is interpreted in terms of the interaction among the component, without properly considering the possible transformation of each individual part.

## Experimental Section

### Chemicals and Materials

The synthesis was carried out using standard airless procedures and commercially available reagents: Benzyl ether (99%), 1-octadecene (90%), oleic acid (OA, 90%), oleylamine (OAm, >70%), iron(III) acetylacetonate (Fe(acac)_3_, 99%), and cobalt(II) chloride anhydrous (CoCl_2_, ≥99%). All starting materials were purchased from Sigma-Aldrich and used without further purification. CoCl_2_ anhydrous was stored inside a glove box.

### Synthesis of Cobalt Ferrite NPs (CFO)

**Scheme 1:**
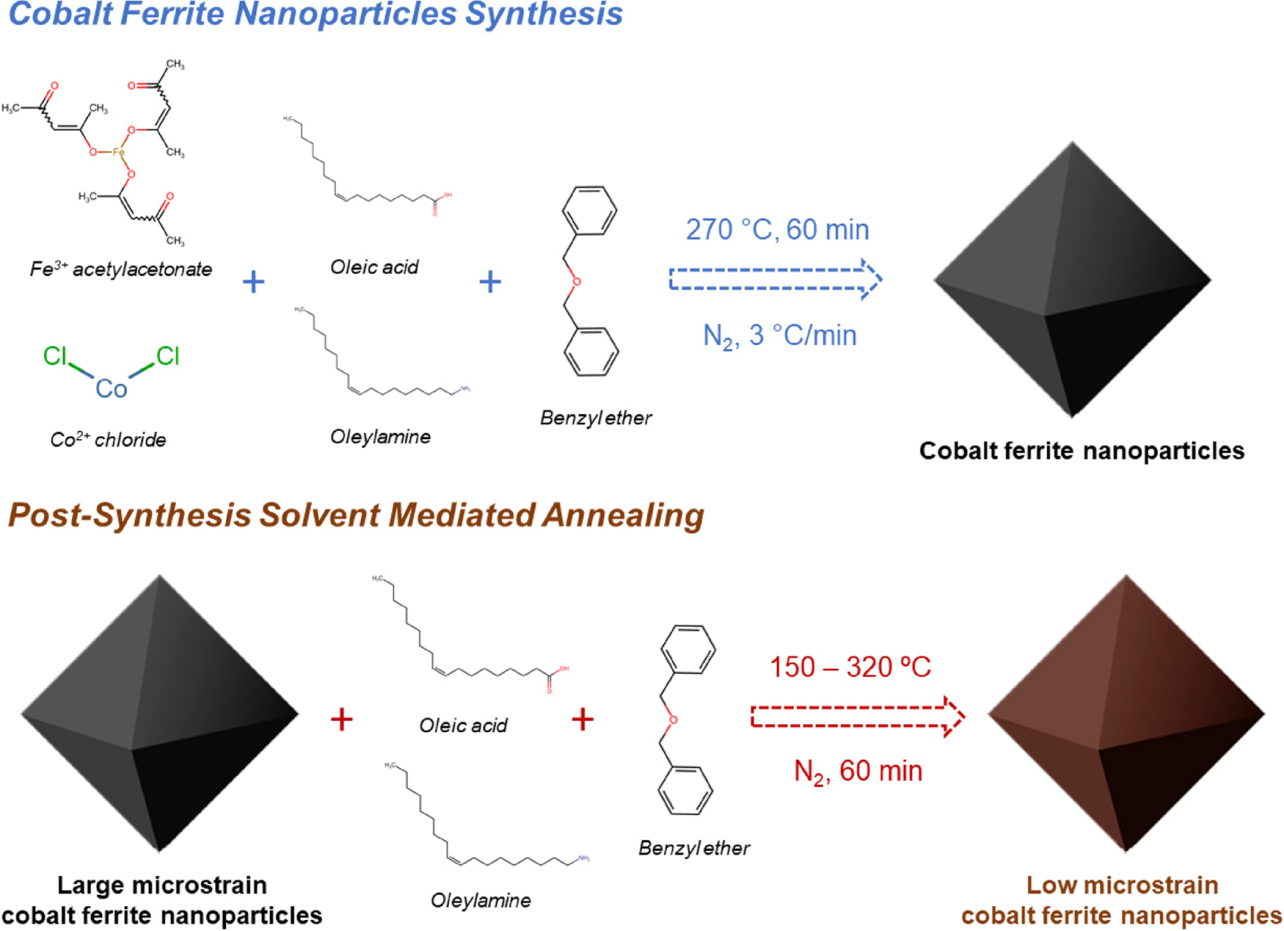
Schematic Representation of the Cobalt Ferrite Nanoparticle Synthesis and the Posterior Solvent-Mediated Annealing

Cobalt ferrite NPs (**CFO**) were synthesized by dissolving 2 mmol of Fe(acac)_3_, 1 mmol of CoCl_2_, 6 mmol of OA, and 6 mmol of OAm in 25 mL of benzyl ether in a 50 mL three-neck round bottomed flask (Scheme 1). Initially, the mixture was degassed bubbling N_2_ at 100 °C for 60 min, and then it was heated up to 270 °C for 60 min with a heating rate of 3 °C/min. During the heating and digestion process, the mixture was exposed to an N_2_ flow. Finally, the flask was removed from the heating mantle and allowed cooling down under an inert atmosphere. The NPs were washed by several cycles of coagulation with ethanol, centrifugation at 5000 rpm, disposal of supernatant solution, and re-dispersion in hexane.

### Solvent-Mediated Annealing Treatment of CFO

Afterward, **CFO** NPs were subjected to further heating process at different temperatures following a procedure commonly used for the growth of a ferrite shell.^8^ 0.025 g of as-prepared NPs were re-dispersed in a solution containing 0.6 mmol of OA, 0.6 mmol of OAm, and 40 mL of benzyl ether or 1-octadecene in a 100 mL three-neck round bottomed flask. The mixture was degassed bubbling N_2_ at 100 °C for 60 min, and then it was heated up to the desired temperature (150, 210, 270, or 320 °C) for 60 min with a heating rate of 3 °C/min. During the heating process, the mixture was exposed to a N_2_ flow. Finally, the flask was removed from the heating mantle and allowed cooling down under an inert atmosphere. The NPs were washed by several cycles of coagulation with ethanol, centrifugation at 5000 rpm, disposal of supernatant solution, and re-dispersion in hexane. In addition, 0.025 g of **CFO** in powder was heated at 3 °C/min up to 210 °C under an N_2_ atmosphere in an oven. The sample was kept at this temperature for 60 min and then cooled down to room temperature.

### Structural, Morphological, and Chemical Composition Characterization

TEM images were obtained using a Philips CM12 microscope with a LaB_6_ filament operated at 100 kV. The NPs were dispersed in hexane and then placed dropwise onto a holey carbon supported grid. The particle size of the different samples and the standard deviation were obtained by calculating the number average by manually measuring the equivalent edge length of >200 octahedral particles from TEM micrographs. Ultrahigh-resolution TEM (UHRTEM) images were acquired at 200 kV on a JEOL JEM-2200FS equipped with a Ω-filter (point resolution 0.19 nm). GPA was performed with the FRWRtools plugin for Digital Micrograph (Gatan, Inc.) on **CFO** and **CFO210** by analyzing 10–15 NPs.^61^ The structure of the NPs was investigated by powder X-ray diffraction (XRD) using a Bruker New D8 ADVANCE ECO diffractometer equipped with Cu Kα radiation. The measurements were carried out in the range of 20–90°, with a step size of 0.01° and a collection time of 1 s. Quantitative analysis of the XRD data was undertaken with a full pattern fitting procedure based on the Rietveld method using the MAUD program.^62^ All the weighted profile *R*-factors (*R*_WP_) obtained from Rietveld analysis are in the range between 0.082 and 0.091. The transition metal content (w/w) in NPs was assessed by using an EDXRF spectrometer Shimadzu EDX-7000.

### Magnetic Measurements

The magnetic properties of the NPs were measured on tightly packed powder samples using a vibrating sample mode (VSM, Quantum Design PPMS) magnetometer with 9 T maximum field. The hysteresis loops were measured at increasing temperatures after field cooling the sample in 5 T from 380 to 5 K. Saturation magnetization has been calculated extrapolating the M vs 1/H at high fields and the effective cubic magnetic anisotropy using the expression *K*_eff_ = *H*_C_*M*_S_/0.64.^8^

^57^Fe Mössbauer spectra were recorded using a ^57^Co/Rh γ-ray source mounted on an electromagnetic transducer with a triangular velocity form, at 12 K in an 8 T field oriented in parallel to the γ-beam. For the analysis of Mössbauer spectra, the program “Mosfit” was used. The hyperfine structure was modeled by a least-squares fitting procedure involving Zeeman sextets composed of Lorentzian lines. To describe the broadening of lines, several magnetic subcomponents were considered where isomer shift, quadrupolar shift, linewidth, and effective field values were left free during the refinement and the intensities of intermediate lines (2, 5) as well; the ratio of intensities of external/internal lines being found systematically equal to 3. The isomer shift (IS) values were referred to α-Fe at 300 K. The samples consisted of a thin layer of about 40 mg of the powdered compound.

## Supporting Information Available

Energy-dispersive X-ray fluorescence; high-resolution transmission electron microscopy; X-ray diffraction data; X-ray diffraction data; Mössbauer spectroscopy; magnetic characterization; and Δ*M* plots (PDF)
