# Correction to “Hardening of Cobalt Ferrite Nanoparticles by Local Crystal Strain Release: Implications for Rare Earth Free Magnets”

**DOI:** 10.1021/acsanm.5c04000

**Published:** 2025-09-12

**Authors:** Beatrice Muzzi, Elisabetta Lottini, Nader Yaacoub, Davide Peddis, Giovanni Bertoni, César de Julián Fernández, Claudio Sangregorio, Alberto López-Ortega

In our original paper, the scale values of the x-axis in panel a of Figure 5 were incorrect. The corrected Figure 5 is provided below.

**Figure 5:**
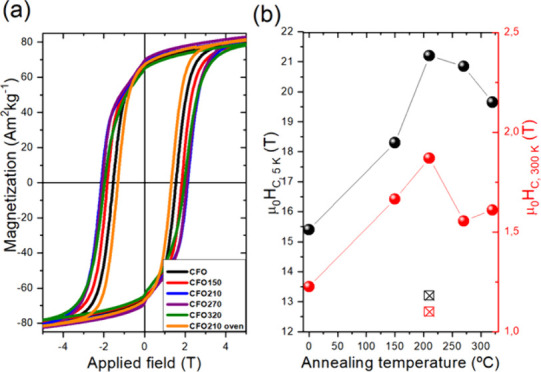
(a) Hysteresis loops for the as-prepared and annealed cobalt ferrite NPs measured at 5 K. (b) Coercive field (μ_0_ *H*_C_) dependence measured at 5 K (black) and 300 K (red) as a function of the annealing temperature (black ballot box with X and red ballot box with X refer to **CFO210-oven**).

