# Supplementary material for: Hardening of Cobalt Ferrite Nanoparticles by Local Crystal Strain Release: Implications for Rare Earth Free Magnets
Source: ACS Appl Nano Mater. 2022 Sep 21;5(10):14871–81. doi: 10.1021/acsanm.2c03161 (PMC9624260; doi:10.1021/acsanm.2c03161)
Supplement: Supplementary file 1 — an2c03161_si_001.pdf [file an2c03161_si_001.pdf]

SUPPORTING INFORMATION

# Hardening of Cobalt Ferrite Nanoparticles by Local Crystal Strain Release: Implications for Rare Earth Free Magnets

*Beatrice Muzzi,[1,2,3] Elisabetta Lottini,[3] Nader Yaacoub,[4] Davide Peddis,[5,6] Giovanni Bertoni,[7] César de Julián Fernández,[8] Claudio Sangregorio,[2,3*] Alberto López-Ortega[3,9,10*]*

lopezortega.alberto@gmail.com, csangregorio@iccom.cnr.it

[1]Department of Biotechnology, Chemistry and Pharmacy, University of Siena 1240, I-53100 Siena, Italy

[2]ICCOM – CNR, I-50019 Sesto Fiorentino (FI), Italy

[3]Department of Chemistry "U. Schiff", University of Florence and INSTM, I-50019 Sesto Fiorentino (FI), Italy

[4]IMMM, Université du Mans, CNRS UMR-6283, F-72085 Le Mans, France

[5]Department of Chemistry and Industrial chemistry, University of Genoa, I-16146 Genova, Italy

[6]ISM – CNR, I-00015 Monterotondo Scalo (RM), Italy

[7]CNR – Istituto Nanoscienze, I-41125 Modena, Italy

[8]IMEM – CNR, I-43124 Parma, Italy

[9]Departamento de Ciencias, Universidad Pública de Navarra, E-31006 Pamplona, Spain

[10]Institute for Advanced Materials and Mathematics, Universidad Pública de Navarra, E-31006 Pamplona, Spain

# Energy Dispersive x-Ray Fluorescence (EDXRF)

**Table S1**. Iron and cobalt (w/w) relative percentage obtained by Energy Dispersive X-Ray Fluorescence (EDXRF) for CFO before and after thermal annealing at different temperatures.

| Samples | Fe % (w/w) | Co % (w/w) |
|---|---|---|
| **CFO** | 87.1(5) | 12.9(5) |
| **CFO150** | 87.2(5) | 12.8(5) |
| **CFO210** | 85.8(5) | 14.2(5) |
| **CFO320** | 85.6(5) | 14.4(5) |

# High-Resolution Transmission Electron Microscopy (HRTEM)

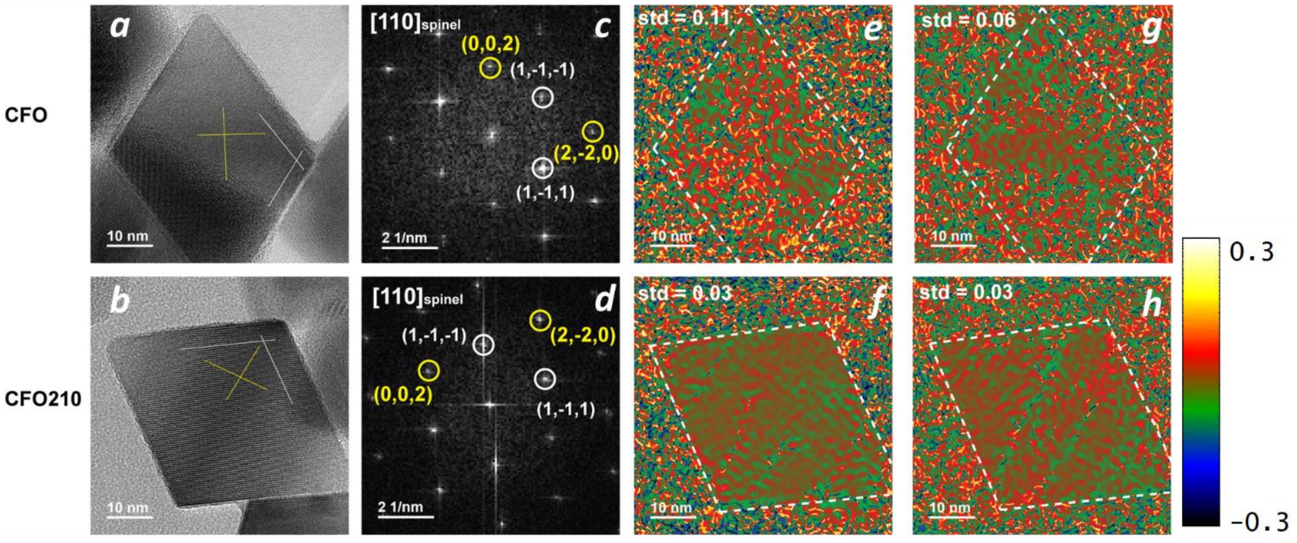


**Figure S1.** HRTEM images of a (a) **CFO** and a (b) **CFO210** NP; FFT analysis from the NP in (a) and (b), respectively, showing the presence of the same crystallographic structure in **CFO** and **CFO210**. The labelled spots are related to crystallographic planes that can be indexed as cubic spinel structure ($Fd\bar{3}m$), in zone axis [110]. Strain maps $\varepsilon_{xy}$ (symmetric shear) obtained by GPA analysis of the NPs (e, g) before and (f, h) after thermal annealing. The maps are calculated from (e, f) $\mathbf{g}_1$ = (002) and $\mathbf{g}_2$ = (2-20) reflections, and from (g, h) $\mathbf{g}_1$ = (1-1-1) and $\mathbf{g}_2$ = (1-11) reflections respectively, and plotted in the relative interval (-0.3, +0.3).

The standard deviation (std) calculated from the strain maps obtained for (111) planes in [011] orientation, decreased from 0.11 for the as-prepared **CFO** (ca. 11% relative variation) to 0.03 for **CFO210** (ca. 3% relative variation). Conversely, a similar std (0.03) was found for (002) and (220) reflections in both the samples. This value can be thus considered as that measured in the case of low disorder/strain at our conditions of imaging (acquisition noise, presence of a support carbon amorphous film below the particles, and residual geometrical aberrations from the objective lens).

## x-Ray diffraction data

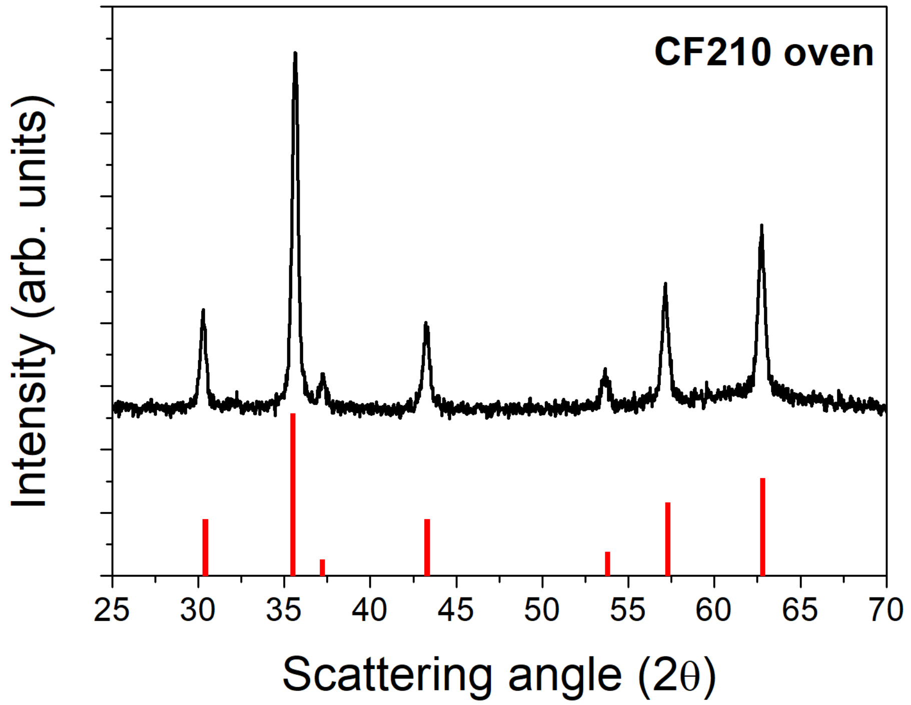


**Figure S2.** XRD pattern for sample annealed in oven, **CFO210-oven** (red bars refer to the position of the diffraction peaks for the cobalt ferrite crystal structure).

## Mössbauer spectroscopy

$^{57}$Fe Mössbauer spectroscopy under intense magnetic field is a powerful tool to investigate magnetic structure of spinel ferrite, allowing a more reliable distinction between A- and B-site components than the zero field spectra, allowing to better determine all the hyperfine parameters, as mean isomer shift ($\delta$), mean quadrupolar shift ($2\varepsilon$), and mean hyperfine field ($B_{hyp}$). The applied field is usually added to the A-site hyperfine field and subtracted from the B-site hyperfine field, $B_{hyp}$, allowing less overlap between the two

components. Furthermore, such spectra can also give information about non-collinearity of the spin structure, mean canting angle ($\theta$).

In the presence of an external magnetic field parallel to the γ- ray direction, the relative areas of the absorption lines give information about the degree of alignment of the magnetization with the applied field. The modelling of the in-field Mössbauer spectrum allows one also the direct estimation of both the effective field ($B_{eff}$) and the angle ($\theta$) of the two types of sites, for an inverse spinel structure, and then their respective hyperfine field $B_{hyp}$ can be calculated according to the relation:

$$B_{hyp}^2 = B_{eff}^2 + B_{app}^2 - 2B_{eff}B_{app}\cos\theta \qquad (1)$$

where $B_{eff}$ is the vectorial sum of the $B_{hyp}$ and the applied field, $B_{app}$, and $\theta$ is the angle between the magnetic field at the nucleus and the γ-ray direction, respectively. For a thin absorber the relative area of the six lines is given by 3:p:1:1:p:3, where:

$$p = \frac{4\sin^2\vartheta}{1+\cos^2\vartheta} \qquad (2)$$

In particular, the experimental results reveal the presence of an outer sub-spectrum (red line in figure 3) with symmetrical and narrow lines corresponding to $Fe^{3+}$ in A-sites according to the hyperfine parameters (Table 2), and an inner sub-spectrum (octahedral sites, blue line in the figure 3) with an asymmetric and complex shape. This complexity comes from the presence of $Fe^{2+}$, which is characterized by a weak hyperfine field and large isomer shift compared with those of $Fe^{3+}$. The description of this in-field sub-spectrum requires a model comprising several components of sextet splitting composed of Lorentzian lines (Figure S3), considering the correlation between isomer shift, effective field, quadripolar shift and angle canting in each component (Table S2).

The mean refined values of the hyperfine parameters correspond to those of $Fe^{3+}$ in A-sites of cobalt ferrite structure[2]. On the other hand, the $<B_{hyf}>$ of the iron species located in octahedral sites (51.2 T) are slightly lower than those of stoichiometric cobalt ferrite, and the mean value of the isomer shift of iron located in octahedral site ($\cong$ 0.5 $mm/s^{-1}$) is higher than that of $Fe^{3+}$ in cobalt ferrite NPs.[4,5] Therefore, the values of hyperfine parameters show the presence of iron ions at intermediate valence between $Fe^{3+}$ and $Fe^{2+}$.[6]

The nonzero intensity of lines 2 and 5 (i.e., $\theta \neq 0$) in the spectra indicates the presence of a non-collinear magnetic structure, with some spins that are not aligned parallel or antiparallel to the external magnetic field.

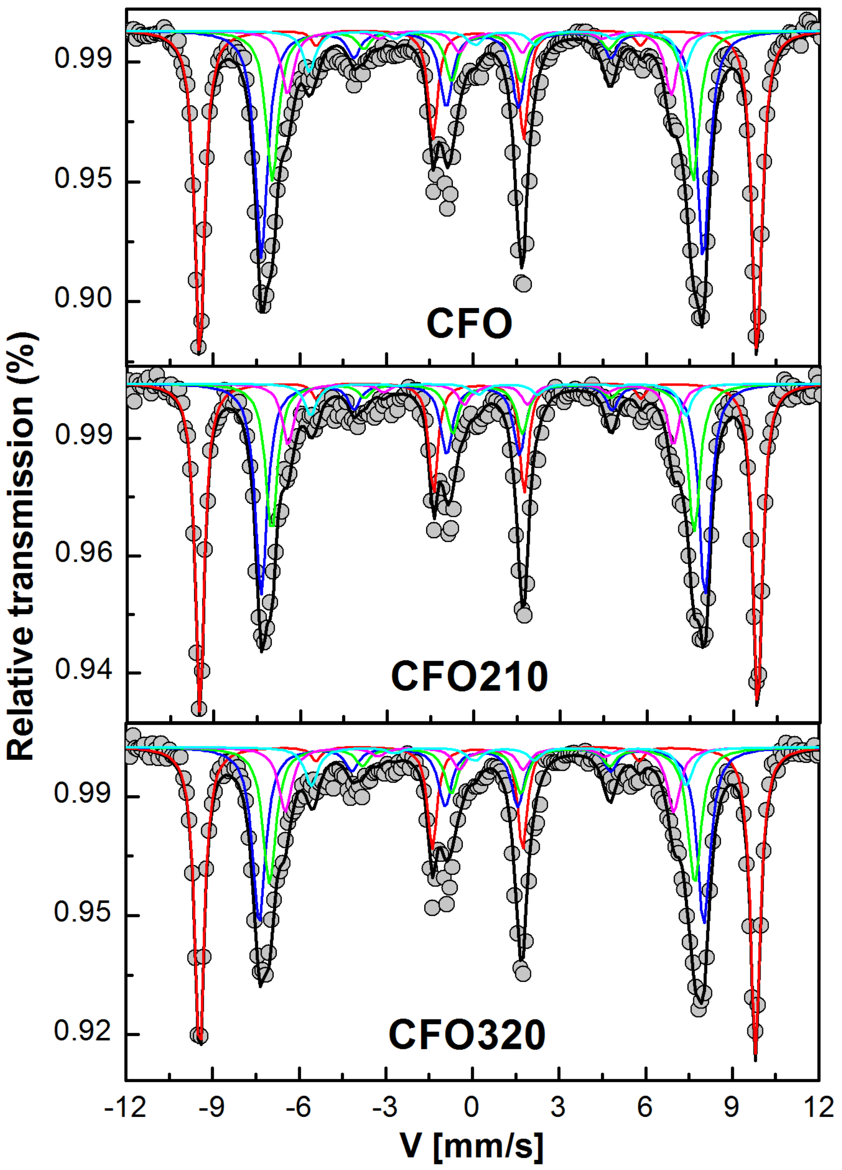


**Figure S3.** Mössbauer spectra measured at 12 K under 8 T magnetic field for samples **CFO**, **CFO210** and **CFO320**. Grey dots and solid lines are experimental and simulated data. Black and red simulated lines are the total spectrum and sub-spectrum corresponding to A site, respectively; blue, green, pink and cyan simulated lines are the sub-spectrum corresponding to $Fe^{3+}$, $Fe^{2+<x<3+}$, $Fe^{2+}$ in B sites, respectively.

**Table S2.** Mean isomer shift (δ), mean quadrupole shift (2ε), mean effective field ($B_{eff}$), mean hyperfine field ($B_{hyf}$), mean canting angle (ϑ) and relative amount of Fe in in the cavities evaluated from fitting Mössbauer spectra (recorded at 12 K applying an 8 T magnetic) of samples **CFO**, **CFO210**, and **CFO320**. Uncertainties on the last digit are given in parentheses. The error for canting angle has been assessed to be 10º.

| **CFO** | | | | | | |
|---|---|---|---|---|---|---|
| **12K 8T** | **δ** | **2ε** | $\mathbf{B_{eff}}$ | $\mathbf{B_{hyp}}$ | **θ** | **%** |
| $Fe^{3+}A$ | 0.36 (1) | 0.00 (1) | 59.7 (1) | 52.0 (1) | 15 | 34 |
| $Fe^{3+}B$ | 0.50 (1) | 0.00 (1) | 47.2 (1) | 54.6 (1) | 23 | 31 |
| $Fe^{!+}B$ | 0.57 (1) | -0.12 (1) | 45.0 (1) | 52.5 (1) | 23 | 20 |
| $Fe^{!+}B$ | 0.61 (1) | -0.4 (1) | 41.0 (1) | 48.5 (1) | 23 | 9 |
| $Fe^{2+}B$ | 1.18 (1) | -0.3 (1) | 40.0 (1) | 33.5 (1) | 32 | 6 |
| **CFO 210** | | | | | | |
| **12K 8T** | **δ** | **2ε** | $\mathbf{B_{eff}}$ | $\mathbf{B_{hyp}}$ | **θ** | **%** |
| $Fe^{3+}A$ | 0.35 (1) | 0.00 (1) | 59.7 (1) | 52.0 (1) | 14 | 34 |
| $Fe^{3+}B$ | 0.50 (1) | 0.00 (1) | 47.4 (1) | 54.8 (1) | 23 | 31 |
| $Fe^{!+}B$ | 0.57 (1) | -0.17 (1) | 45.1 (1) | 52.6 (1) | 23 | 21 |
| $Fe^{!+}B$ | 0.67 (1) | -0.52 (1) | 41.1 (1) | 48.6 (1) | 23 | 9 |
| $Fe^{2+}B$ | 1.17 (1) | -0.31 (1) | 40.0 (1) | 32.5 (1) | 19 | 6 |
| **CFO320** | | | | | | |
| **12K 8T** | **δ** | **2ε** | $\mathbf{B_{eff}}$ | $\mathbf{B_{hyp}}$ | **θ** | **%** |
| $Fe^{3+}A$ | 0.35 (1) | 0.00 (1) | 59.5 (1) | 51.8 (1) | 15 | 34 |
| $Fe^{3+}B$ | 0.49 (1) | 0.00 (1) | 47.5 (1) | 54.8 (1) | 25 | 28 |
| $Fe^{!+}B$ | 0.58 (1) | -0.14 (1) | 45.4 (1) | 52.7 (1) | 25 | 22 |
| $Fe^{!+}B$ | 0.63 (1) | -0.41 (1) | 41.5 (1) | 48.8 (1) | 25 | 10 |
| $Fe^{2+}B$ | 1.18 (1) | -0.17 (1) | 40.0 (1) | 33.0 (1) | 26 | 6 |

## Magnetic characterization

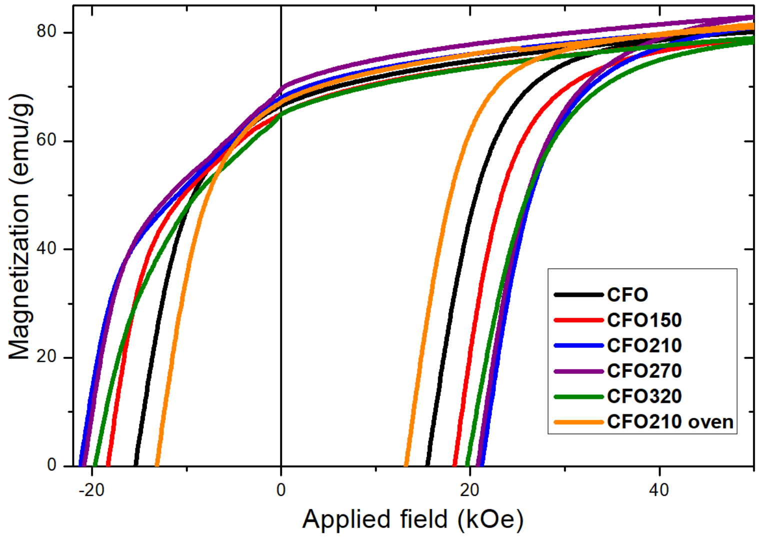


**Figure S4.** Magnification of the hysteresis loops for the as prepared and annealed cobalt ferrite NPs measured at 5 K. Samples treated at higher temperature (**CFO270** and **CFO320**) display an almost linear dependence from 2 to 9 kOe. This effect can be associated with the chemical composition inhomogeneity produced by the high temperatures used to prepare these two samples, which modifies the cell parameter and the microstrain in the nanostructure.

## ΔM Plots

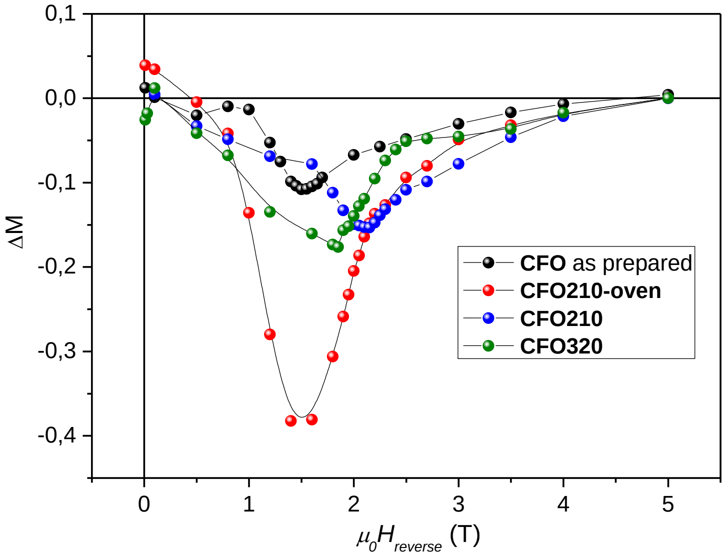


**Figure S5.** Henkel plot of samples **CFO** (black dots), **CFO210** (blue dots), **CFO 320** (green dots) and **CFO210-oven** (red dots)

The field dependence of remanent magnetization was measured using the IRM (Isothermal Remanent Magnetization) and DCD (Direct Current Demagnetization) protocols. According to the IRM protocol, the samples, in the demagnetized state, were cooled in a zero magnetic field down to 5 K. At this temperature, a small external field was applied for 10 s, then switched off, and finally, the remanence ($M_{IRM}$) was measured. The process was repeated, increasing the field in steps up to 5 T. In a DCD measurement, the initial state was the magnetically saturated one. After cooling the sample at 5 K, an external field of -5 T was applied for 10 s, then it was turned off and the remanence ($M_{DCD}$) was measured. As in IRM, a small external field in the opposite direction to magnetization was applied for 10 s and then switched off. Finally, the remanent magnetization was measured. This was repeated increasing the field up to +5 T. The analysis of the remanent magnetization curves measured by *IRM* and *DCD* protocols (**Figure S3**) allowed us to investigate the interaction regime among particles. For an assembly of non-interacting single-domain particles with uniaxial anisotropy and magnetization reversal by coherent rotation, the two remanence curves are related via the Wohlfarth equation [1]:

$$m_{DCD}(H) = 1 - 2m_{IRM}(H) \qquad (1)$$

where $m_{DCD}(H)$ and $m_{IRM}(H)$ represent the reduced terms $M_{DCD}(H)/M_{DCD(5T)}$ and $M_{IRM}(H)/M_{IRM(5T)}$, and $M_{DCD(5T)}$ *and* $M_{IRM(5T)}$ are the remanence values for the *DCD* and *IRM* curves for a reverse field of 5 T, respectively. Kelly et al. [2] rewrote the Wohlfarth relation to explicitly reveal deviations from a non-interacting case:

$$\Delta M = m_{DCD}(H) - 1 + 2m_{IRM}(H) \qquad (2)$$

Negative $\Delta M$ are usually taken as indicative of the prevalence of demagnetizing (e.g., dipole-dipole) interactions; positive values are attributed to interactions promoting the magnetized state (e.g., direct exchange interactions). Qualitatively, the intensity of such deviation ($I_{\Delta M}$) can be considered proportional to the strength of the interparticle interactions.[3]

## Magnetic characterization

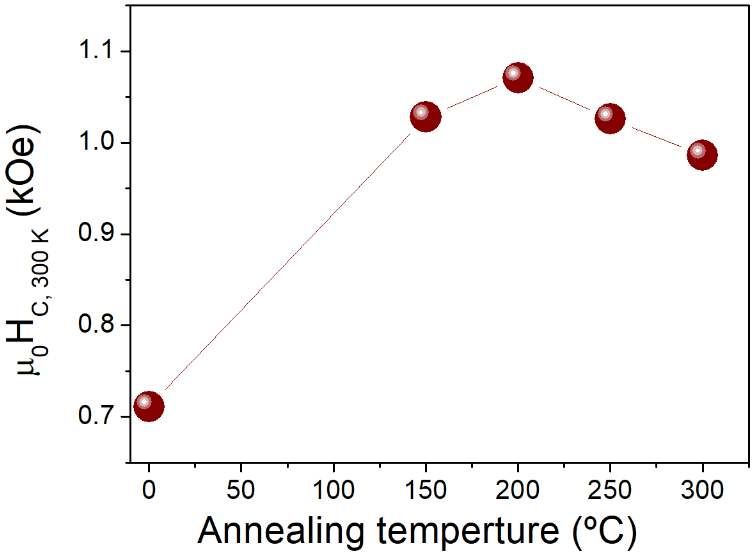


**Figure S6.** Coercive field ($H_C$) dependence measured at 300 K as a function of the annealing temperature for sample **CFO_2_#**. Inset depicts low resolution TEM image and the corresponding particle size histogram.

## References


1 J. Santoyo Salazar, L. Perez, O. de Abril, L. Truong Phuoc, D. Ihiawakrim, M. Vazquez, J.-M. Greneche, S. Begin-Colin, G. Pourroy, J. S. Salazar, O. De Abril and L. T. Phuoc, *Chem. Mater.*, 2011, **23**, 1379–1386.

2 P.E. Kelly, K. O'Grady, P.I. Mayo, Int. Magn. Conf. 1989, **25**, 3881–3883.

3 M. Artus, L. Ben ahar, F. Herbst, L. Smiri, F. Villain, N. Yaacoub, J.-M. Greneche, S. Ammar and F. Fiévet, *J. Phys. Condens. Matter*, 2011, **23**, 506001.
